# Association between dairy products intake and frailty transitions in older adults: The InCHIANTI cohort study

**DOI:** 10.1016/j.jnha.2025.100482

**Published:** 2025-01-14

**Authors:** Nicole Hidalgo-Liberona, Tomás Meroño, Raul Zamora-Ros, Caterina Trevisan, Massimiliano Fedecostante, Stefania Bandinelli, Luigi Ferrucci, Antonio Cherubini, Cristina Andres-Lacueva

**Affiliations:** a Biomarkers and Nutrimetabolomics Laboratory, Department of Nutrition, Food Sciences and Gastronomy, Faculty of Pharmacy and Food Sciences, University of Barcelona, 08028 Barcelona, Spain; b CIBER de Fragilidad y Envejecimiento Saludable (CIBERFES), Instituto de Salud Carlos III, 28029 Madrid, Spain; c Unit of Nutrition and Cancer, Cancer Epidemiology Research Programme, Catalan Institute of Oncology (ICO), Bellvitge Biomedical Research Institute (IDIBELL), 08908 L’Hospitalet de Llobregat, Spain; d Department of Medical Sciences, University of Ferrara, 44121 Ferrara, Italy; e Aging Research Center, Karolinska Institutet, 17177 Stockholm, Sweden; f Geriatria, Accettazione Geriatrica e Centro di Ricerca per l’Invecchiamento, IRCCS INRCA, 60127 Ancona, Italy; g Geriatric Rehabilitation Unit, Azienda Sanitaria Firenze, 40125 Florence, Italy; h Clinical Research Branch, National Institute on Aging, NIH, 21224 Baltimore, MD, United States; i Deparment of Clinical and Molecular Sciences, DISCLIMO, Università Politecnica Delle Marche, 60121 Ancona, Italy

**Keywords:** Frailty, Nutritional status, Dairy products, Yogurt, Cohort

## Abstract

**Objective:**

To evaluate the association between dairy products consumption and the probability of frailty transitions in community-dwelling older adults.

**Design:**

Longitudinal study.

**Setting and participants:**

We included 863 community-dwelling participants ≥65 years from the Chianti region in Italy.

**Mesurements:**

Habitual dietary intake of dairy products (*i.e.*, milk, yogurt, and cheese) was assessed in daily servings using a validated food frequency questionnaire (FFQ) at baseline, 3-, 6-, and 9-years of follow-up. Frailty status at each visit was defined using the Fried criteria, and the probability of transitions between different frailty status and death was assessed through multistate models. The associations between dairy product intakes and frailty transitions during the 9-year period were expressed as hazard ratios (HRs) derived from proportional intensity models.

**Results:**

The mean age at baseline was 74 ± 7 years and 46% of the participants were male. There were no statistically significant associations between the consumption of total, fermented, or non-fermented dairy products and the probabilities of transition from robust or from pre-frail to any of the other frailty conditions or to death. Conversely, a direct association between the consumption of fermented dairy products and the probability of transition from frail to pre-frail was observed in a model adjusted for age, sex, and energy intake (HR_per serving/day_ = 1.90, 95%CI 1.12−3.22). This association was primarily related to yogurt consumption (HR_per serving/day_ = 4.07, 95%CI 1.38−12.02), as the association with cheese consumption was not significant (HR_per serving/day_ = 1.57, 95%CI 0.91−2.71). In the fully adjusted model, only the association between yogurt consumption and frail to pre-frail transition remained statistically significant (HR_per serving/day_ = 3.68, 95%CI 1.10−12.31).

**Conclusion:**

Dairy products, such as milk, yogurt, and cheese, are unlikely to play a predominant role in frailty development in an Italian community-dwelling older population. However, it is advisable to maintain a moderate consumption of dairy products, especially fermented ones, as part of a well-balanced diet to promote healthy aging.

## 1 Introduction

Frailty is a geriatric condition characterized by an increased vulnerability, which is accompanied by a high risk of illness, falls, hospitalizations, disability, and death. Nevertheless, it is a dynamic process that can be reversed, particularly in the early stages [[1], [2], [3]]. Although frailty can be observed in middle-aged adults, it is closely linked to aging, and therefore, its prevalence increases as the population ages [4]. Several methods to diagnose frailty have been developed [5], and the frailty phenotype proposed by Fried et al. [1] is one of the most commonly used to identify frail older subjects, easily allowing the comparison of our findings with other epidemiological studies and the translation to clinical care. It considers five components: shrinking or unintentional weight loss, weakness (low grip strength), poor endurance or exhaustion, slowness (slow walking speed), and low physical activity [1]. Thus, the Fried’s frailty phenotype is able to capture aspects related to both muscle and bone health, including muscle strength and performance.

Different health conditions and lifestyle habits, including inadequate nutritional status, contribute to the development of frailty [[6], [7], [8]]. Inadequate energy intake may cause undernutrition and micronutrient deficiencies, leading to frailty in older adults [3,7,9]. Indeed, previous cross-sectional analyses have already shown that a low nutrient intake was associated with frailty in the InCHIANTI [*Invecchiare in Chianti* (Aging in Chianti)] and in a US-based study [10,11]. Likewise, high protein intake was inversely associated with frailty status in older adults [12,13]. Therefore, dietary habits seem to have a relevant role in frailty development. Several studies have found that a high intake of orange juice [14], fruits and vegetables [[15], [16], [17]], and fish [17], as well as a high adherence to healthy dietary patterns [[18], [19], [20]] were associated with lower frailty risk.

Dairy products are a complex source of nutrients, and there is an increasing interest in their potential health effects. They are a dietary source of protein and micronutrients, such as calcium, phosphorus, vitamin A, vitamin D, riboflavin, vitamin B12, potassium, zinc, choline, magnesium, and selenium. The 2020–2025 Dietary Guideline for Americans (DGA) recommends 3 daily servings of dairy products, particularly fat-free or low-fat alternatives, such as milk, cheese, and/or yogurts, for subjects 9 years and older [21]. Moreover, recent studies have shown that higher intakes of dairy products were associated with weight loss and lower risk for type 2 diabetes [22], colorectal cancer [23], cardiovascular disease, and overall mortality [24]. Despite these results, the epidemiological evidence is still inconsistent regarding their relationship with frailty risk in older adults [[25], [26], [27], [28], [29]].

In the current prospective study, we examined the association between the habitual intake of dairy products and the probability of experiencing frailty transitions in older adults from the InCHIANTI study during a 9-years period. As dairy products are nutrient dense sources of proteins and some micronutrients, they may help to maintain/enhance both muscle and bone mass [30,31], reducing sarcopenia and osteoporosis, which are related to frailty [32]. Therefore, we hypothesize that a higher intake of dairy products would be associated with a lower frailty risk and/or a higher probability of frailty transitions towards robustness.

## 2 Methods

This study is developed and reported according to the Strengthening the Reporting of Observational Studies in Epidemiology-Nutritional Epidemiology (STROBE-NUT) checklist (see Supplementary Table S1: STROBE-nut statement) [33].

### 2.1 Population

**Fig. 1:**
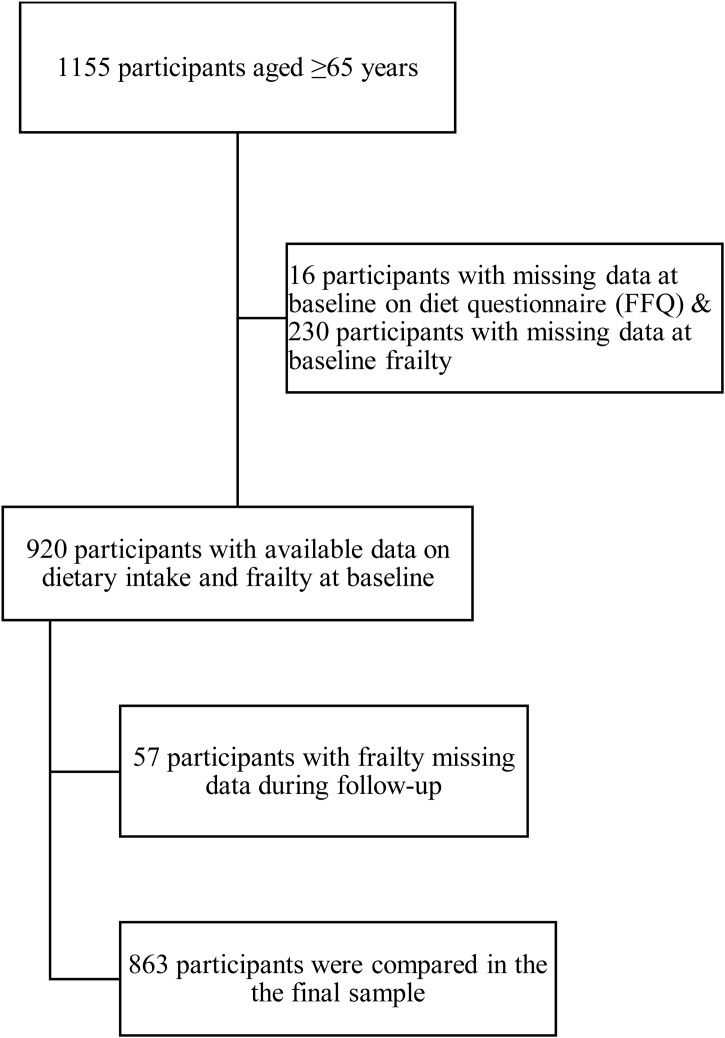
Flowchart of participants at period of the study.

The InCHIANTI study is a prospective cohort of randomly selected older adults conducted in two towns in the Tuscany countryside (Greve in Chianti and Bagno a Ripoli) in Italy. Further details of the study design have been described elsewhere [34]. The study population includes 1155 women and men older than 65 who agreed to participate. We excluded participants who had missing data in the food frequency questionnaire (FFQ) (*n* = 16), and frailty data at recruitment (*n* = 219). For the longitudinal analysis, we excluded 57 participants who were lost at follow-up after the baseline assessment (Fig. 1). For the current analysis, data was available for 863 Italian older adults (total observations = 2510). The ethical committee of the Italian National Institute of Research and Council of Aging examined and approved the study protocol. All participants provided written informed consent.

### 2.2 Dietary assessment

Habitual food intake was assessed at baseline and 3, 6, and 9 years after enrollment, using the Italian version of the FFQ developed and validated in the European Prospective Investigation into Cancer and Nutrition (EPIC-Italy) study [35]. Participants reported the frequency of consumption and usual portion size of 198 food items. Total dairy product consumption (in servings per day) was estimated as the sum of three food items: yogurt, cheese, and milk. Fermented dairy products were calculated as the sum of cheese and yogurt, while non-fermented dairy products only considered milk. Dairy product servings were established according to the different FFQ items; for instance, the portion size for hard and semi-hard cheeses was 65 g, and 100 g for soft and fresh cheese [[1], [2], [3]]. The intakes of total energy (kcal/day), alcohol (g/day), total protein (g/day), and other nutrients were calculated using an Italian food composition database [34]. Missing dietary data during the follow-up (3.4%) were imputed by the Last Observation Carried Forward (LOCF).

### 2.3 Assessment of frailty syndrome

We used the operational definition of frailty phenotype developed by Fried et al. [1]. It was based on 5 components: unintentional weight loss, exhaustion, low physical activity, slow walking speed, and muscle weakness. Briefly, shrinking or unintentional weight loss was self-reported and defined as involuntary weight loss (not due to diet or exercise) >4.5 kg in the prior year. Weakness was defined as grip strength measured using a handheld dynamometer (Nicholas Muscle Tester, Sammon Preston, Inc., Chicago, IL) in the lowest sex-specific quintile at baseline and follow-up visits. Low physical activity was defined as either complete inactivity or spending <1 h/week of low-intensity activities in a physical activity questionnaire developed and validated for the InCHIANTI study [36]. Self-reported exhaustion or poor endurance was assessed using the statement “I felt that everything was an effort” from the Center for Epidemiological Studies–Depression scale (CES–D), a validated version for the Italian population [37]. Slowness was defined as the time to walk 4.57 m or 15 feet (average of two repetitions) in the lowest sex-specific quintile. Frail, pre-frail, and robust subjects were defined as those having three or more, at least one, and none of these criteria, respectively [1]. Frailty and its components were evaluated at baseline and 3-, 6-, and 9-year visits.

### 2.4 Covariate assessment

Trained physicians conducted a standardized interview and clinical examination. Personal information such as age, sex, and lifestyle data were collected using standardized questionnaires. Education level was assessed as years of education. Self-reported smoking habits were categorized as never, former, and current smokers. Height and weight were measured using standard procedures, and body mass index (BMI) was afterwards calculated. Physical activity data was collected using a structured questionnaire developed and validated for the InCHIANTI study. Participants were categorized as sedentary-light physical activity (none or light-intensity physical activity <2–4 h/wk), or moderate-high physical activity (light-intensity activity >4 h/week or moderate-intensity activity 1–2 h/week; *i.e.*, swimming) [36]. Functional status was evaluated using Katz’s Activities of Daily Living (ADL), and ADL disability was defined as disabled ADL ≥ 1. Cognitive performance was assessed by the Mini-Mental State Examination (MMSE), and depression mood was evaluated using the Centre for Epidemiologic Studies Depression Scale (CES-D). Chronic diseases were ascertained by combining information from self-reported medical diagnoses, pharmacological treatments, medical history, clinical examination, and blood tests. Comorbidities considered in this analysis were cardiovascular diseases (including acute myocardial infarction, stroke, angina pectoris, and peripheral arterial disease), diabetes mellitus, cancer, cognitive impairment, and chronic kidney disease.

### 2.5 Statistical analysis

Participants were classified into tertiles according to their usual daily intake of dairy products (servings per day) for descriptive analysis. Baseline characteristics of subjects were reported as means and standard deviations (SD) for continuous variables with a normal distribution, median and 10th and 90th percentiles for continuous variables with a skewed distribution, and number of participants (*n*) and percentages for categorical variables. Baseline characteristics between subjects classified by tertiles of dairy consumption were compared using parametric ANOVA, non-parametric Kruskall-Wallis, and Chi-square tests, as appropriate.

The associations between the consumption of total, fermented, and non-fermented dairy products (as continuous variables in servings per day) and probabilities of frailty transitions during the 9 years of the study were tested using proportional intensity models [38]. Associations were described as hazard ratios (HRs) and 95% confidence intervals (95% CI). To facilitate the convergence of the analyses, we set a discrete-time multistate model. The analyses were firstly adjusted for age, sex, and total energy intake (Model 1) and secondly, for additional potential confounders, including: years of schooling, smoking habits, alcohol intake, cardiovascular disease, diabetes mellitus, cancer, cognitive impairment, and chronic kidney disease). Confounders were selected based on the rationale linking them to both the exposures and the outcome of interest. To better clarify the role of each included covariate, we have illustrated the causal diagram [39] in Supplementary Fig. S1. In order to verify the fitness and robustness of the multistate analysis, first, we assessed and compared log-likelihood and AIC parameters with that of baseline models (*i.e.* multinomial logistic regression); and second, we performed a 5-fold cross-validation of the model and computed the average log-likelihood across folds, obtaining similar values to that obtained in the total sample (data not shown).

The analyses were computed using the IBM SPSS 26.0 (SPSS Inc., Chicago, IL) and R 4.1.3 (R foundation, Vienna).

## 3 Results

### 3.1 Baseline characteristics

The baseline characteristics for the 863 participants are presented in Table 1. The mean age of participants was 74 ± 7 years, and 45% were men. Regarding socio-demographics and lifestyle habits, 14% of the participants were current smokers, 39% reported moderate-high physical activity, and had, on average, 6 ± 3 years of education. Moreover, 48% of the participants had hypertension, 22% had cardiovascular diseases, and 14% had diabetes mellitus. In comparison, participants excluded due to missing data at baseline (*n* = 292) were significantly older (81 ± 8 *vs* 74 ± 7 years), had more years of education (13 *vs* 6), had higher rates of disability in activities of daily living (ADL) (35% *vs* 3%), depression mood (51% *vs* 30%), and a higher prevalence of dementia (27% *vs* 2%) (*p* < 0.001).

**Table 1:** Baseline characteristics of the InCHIANTI population according to tertiles of dairy product consumption.

Demographics	All (*n* = 863)	Tertile 1 (*n* = 280)	Tertile 2 (*n* = 288)	Tertile 3 (*n* = 287)	*P-value*
		<2.1 servings per day	2.1–3.1 servings per day	>3.1 servings per day	
Age, mean ± SD, y	74 ± 7	73 ± 6	74 ± 7	74 ± 7	0.16
Male, *n* (%)	393 (45.5)	136 (44.0)	145 (47.2)	129 (42.4)	0.65
Education, mean ± SD, y	5.6 ± 3.3	5.7 ± 3.5	5.6 ± 3.3	5.4 ± 3.1	0.93

Behaviour-related variables					
Body Mass Index, mean ± SD, kg/m²	27.6 ± 4.1	27.6 ± 4.0	27.9 ± 4.2	27.2 ± 4.1	0.07
Current smoker, *n* (%)	123 (14.3)	36 (12.6)	47 (16.3)	40 (13.8)	0.79
Physical activity, *n* (%)					0.36
Sedentary-light	527 (61.1)	177 (62.1)	167 (57.8)	183 (63.3)	
Moderate-high	336 (38.9)	108 (37.9)	122 (42.2)	106 (36.7)	
Cognitive impairment, MMSE, *n* (%)	208 (24.1)	75 (26.3)	65 (22.5)	68 (23.5)	0.54
ADL disability, *n* (%)	33 (3.8)	12 (4.2)	15 (5.2)	6 (2.1)	0.14
Depressed mood, CES-D ≥16, *n* (%)	259 (30.0)	84 (29.5)	82 (28.4)	93 (32.2)	0.59

Dietary intake, median (p10–90th)					
Energy, (kcal/d)	1876 (1298−2657)	1665 (1101−2480)	1875 (1345−2670)	2064 (1475−2810)	<0.001
Protein, (g/day)	74.3 (51.5−102.9)	63.7 (43.4−91.8)	74.5 (53.2−101.1)	83.0(61.1−110.4)	<0.001
Alcohol, (g/day)	7.6 (0.0−40.8)	6.1 (0.0−41.4)	9.6 (0.0−49.6)	6.0 (0.0−32.9)	0.05
Calcium, (mg/day)	801 (494−1192)	567 (393−833)	814 (600−1082)	985 (768−1430)	<0.001
Phosphorous, (mg/day)	1165 (817−1616)	990 (691−1390)	1165 (868−1547)	1327 (1015−1806)	<0.001
Vitamin D, (mcg/day)	1.7 (1.0−2.8)	1.6 (0.9−2.8)	1.7 (1.0−2.8)	1.8 (1.1−3.0)	0.001
Potassium (mg/day)	2867 (2022–3941)	2600 (1788–3995)	2888 (2060–3998)	3029 (2218–4064)	<0.001
Fruits, (g/day)	274.9 (144.3−466.8)	260.2 (132.3−477.3)	282.7 (146.8−457.0)	279.1 (153.4−478.8)	0.21
Vegetables, (g/day)	137.1 (66.5−302.8)	134.4 (65.5−304.3)	138.3 (67.5−286.1)	138.9(68.3−305.6)	0.67

Dairy products intake (servings per day)					
Milk	1.0 (0.3−1.1)	0.3 (0.0−1.0)	1.0 (0.0−1.0)	1.0 (1.0−2.0)	<0.001
Yogurt	0.0 (0.0−0.3)	0.0 (0.0−0.1)	0.0 (0.0−0.3)	0.0 (0.0−0.7)	<0.001
Cheese	1.7 (0.4−2.6)	0.6 (0.2−1.6)	1.7 (1−2.5)	2.3 (1.7−3.0)	<0.001
Fermented dairy products	1.8 (0.5−2.7)	0.7 (0.2−1.6)	1.8 (1.1−2.5)	2.4 (1.9−3.2)	<0.001
Non-fermented dairy products	1.0 (0.1−1.5)	0.5 (0.2−1.1)	1.0 (0.7−1.4)	1.1 (1.0−2.1)	<0.001
Total dairy products	2.7 (1.8−3.4)	1.5 (0.6−2.0)	2.7 (2.2−3.0)	3.6 (3.2−4.6)	<0.001

Frailty Phenotype, *n* (%)					0.82
Robust	447 (51.8)	144 (50.5)	158 (54.7)	145 (50.2)	
Pre-Frail	338 (39.2)	115 (40.4)	107 (37.0)	116 (40.1)	
Frail	78 (9.0)	26 (9.1)	24 (8.3)	28 (9.7)	

Frailty Symptoms, *n* (%)					
Weight loss	43 (5.0)	13 (4.6)	13.0(4.5)	17 (5.9)	0.69
Exhaustion	158 (18.3)	53 (18.6)	54 (18.7)	51 (18.3)	0.71
Low physical activity	140 (16.2)	46 (16.1)	48 (16.6)	46 (15.9)	0.97
Slowness	187 (21.7)	58 (21.4)	55 (19.0)	74 (25.6)	0.13
Muscle weakness	172 (19.9)	62 (21.8)	52 (18.0)	58 (20.1)	0.53

Diseases and conditions, *n* (%)					
Hypertension	415 (48.1)	139 (48.8)	148 (51.2)	128 (44.3)	0.24
Cardiovascular diseases	186 (21.6)	63 (22.1)	64 (22.1)	59 (20.4)	0.84
Diabetes mellitus	117 (13.6)	42 (14.7)	36 (12.5)	39 (13.5)	0.72
Diagnosis of dementia	15 (1.7)	4 (1.4)	4 (1.4)	7 (2.4)	0.55
Cancer	52 (6.0)	23 (8.1)	14 (4.8)	15 (5.2)	0.21
Renal chronic disease	181 (30.0)	51 (25.4)	63 (30.4)	67 (34.2)	0.16

*Notes*: Statistical comparisons are from Kruskall-Wallis or Chi-Square tests as appropriate. ADL = Activities of Daily Living; CES-D = Center for Epidemiologic Studies Depression Scale; IADL = Instrumental Activities of Daily Living; MMSE = Mini-Mental State Examination.

The median intake (p10th- p90th) of total dairy products was 2.7 (1.8–3.4) servings per day, and the main contributors were cheese [1.7 (0.4–2.6) servings per day], milk [1.0 (0.3–1.1) servings per day], and yogurt [0.0 (0.0–0.3) servings per day]. Participants in the highest tertile of total dairy product consumption (>3.1 servings per day) were more likely to have a higher intake of total energy, total protein, calcium, phosphorous, potassium, and vitamin D than those in the lowest tertile (<2.1 servings per day) (*p* < 0.001). There were no differences in other lifestyle factors and comorbidities between tertiles of dairy consumption (Table 1).

### 3.2 Dairy intake and frailty

Participants at baseline were mostly robust (52%) and pre-frail (39%), while a few subjects were classified as frail (9%). The most prevalent frailty component was slowness (21.7%), followed by muscle weakness (19.9%), exhaustion (18.3%), low physical activity (16.2%), and unintentional weight loss (5.0%).

**Fig. 2:**
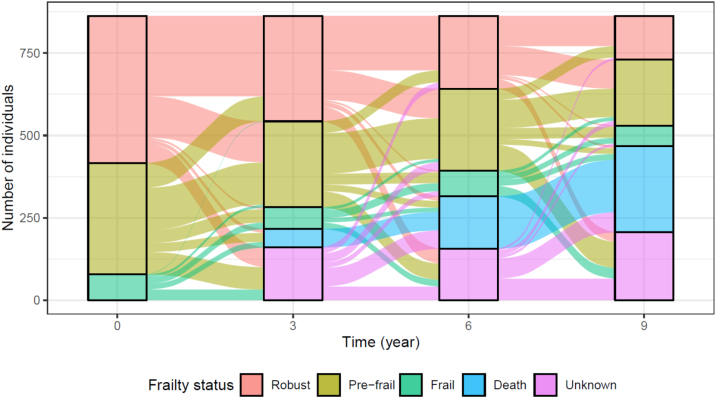
Frailty status during study follow-up. *Notes*: The category “Unknown” includes those either with missing data on frailty or with who did not participate at the specific assessment. (Number of participants = 863, number of observations = 2510).

Fig. 2 shows the transitions between frailty status and death during the 9-years of the study (for the numeric values, see Supplementary Table S2). Probabilities for transitions between robust, pre-frail, and frail status at the different evaluations are reported in Supplementary Table S3. There were no statistically significant associations between the consumption of total, fermented, or non-fermented dairy products and the probabilities of transition from robust or from pre-frail to either of the other frailty conditions or death (Table 2). Conversely, a direct association between the consumption of fermented dairy products and the probability of transition from frail to pre-frail was observed (HR_per serving/day_ = 1.90, 95%CI 1.12−3.22) (Model 1, Table 2). This association was primarily related to yogurt consumption (HR_per serving/day_ = 4.07, 95%CI 1.38−12.02), as the association with cheese consumption was not statistically significant (Table 2). In the fully adjusted model, only the association between yogurt consumption and frail to pre-frail transition remained statistically significant (HR_per serving/day_ = 3.68, 95%CI 1.10−12.31) (Model 2, Table 2).

**Table 2:** Association between dairy product consumption and transitions’ probabilities.

	HR (95%CI) of transition								
	From robust to			From pre-frail to			From frail to		
	Pre-frail	Frail	Died	Robust	Frail	Died	Robust	Pre-frail	Died
Model 1									
Total dairy product	0.98 (0.9−1.07)	1.18 (0.89−1.57)	0.71 (0.45−1.14)	0.96 (0.82−1.12)	1.14 (0.97−1.34)	1.04 (0.79−1.38)	–	1.27 (0.97−1.66)	0.88 (0.66−1.18)
Fermented dairy product	1.06 (0.93−1.2)	1.11 (0.67−1.84)	0.67 (0.37−1.22)	0.93 (0.76−1.15)	1.19 (0.93−1.52)	1.03 (0.69−1.54)	–	**1.90 (1.12−3.22)**	0.86 (0.56−1.32)
Yogurt intake	1.03 (0.74−1.42)	0.76 (0.18−3.23)	0.12 (0−4.76)	1.33 (0.8−2.21)	0.94 (0.48−1.83)	0.86 (0.3−2.48)	–	**4.07 (1.38−12.02)**	1.61 (0.44−5.87)
Cheese intake	1.07 (0.93−1.23)	1.19 (0.68−2.07)	0.76 (0.4−1.42)	0.89 (0.72−1.1)	1.22 (0.94−1.58)	1.06 (0.69−1.63)	–	1.57 (0.91−2.71)	0.83 (0.53−1.29)
Non-fermented dairy product	0.91 (0.8−1.04)	1.22 (0.91−1.65)	0.81 (0.39−1.7)	0.98 (0.78−1.22)	1.09 (0.87−1.36)	1.06 (0.74−1.54)	–	1.05 (0.72−1.54)	0.93 (0.66−1.31)

Model 2									
Total dairy product	0.97 (0.90−1.06)	1.19 (0.90−1.58)	0.67 (0.41−1.11)	0.93 (0.79−1.09)	1.14 (0.96−1.35)	0.98 (0.73−1.32)	–	1.21 (0.91−1.61)	0.90 (0.64−1.25)
Fermented dairy product	1.06 (0.93−1.21)	1.12 (0.67−1.88)	0.70 (0.37−1.32)	0.89 (0.72−1.09)	1.19 (0.93−1.52)	0.98 (0.64−1.51)	–	1.71(0.92−3.19)	0.77 (0.47−1.27)
Yogurt intake	0.97 (0.68−1.37)	0.89 (0.21−3.66)	0.17 (0.00−5.09)	1.26 (0.74−2.13)	0.93 (0.47−1.85)	0.91 (0.29−2.84)	–	**3.68 (1.10−12.31)**	1.43 (0.34−6.04)
Cheese intake	1.08 (0.93−1.24)	1.17 (0.67−2.06)	0.78 (0.40−1.51)	0.85 (0.69−1.05)	1.22 (0.94−1.58)	0.98 (0.62−1.55)	–	1.32 (0.71−2.46)	0.73 (0.44−1.23)
Non-fermented dairy product	0.90 (0.78−1.03)	1.21 (0.89−1.65)	0.62 (0.27−1.48)	0.99 (0.79−1.23)	1.09 (0.87−1.38)	0.98 (0.66−1.46)	–	1.07 (0.75−1.50)	0.98 (0.69−1.39)

Model 1 is adjusted for age, sex, and total energy intake. Model 2 is further adjusted for years of schooling, smoking habits, alcohol intake, cardiovascular disease, diabetes mellitus, cancer, chronic kidney disease, and cognitive impairment. *Abbreviations*: HR, hazard ratio, 95%CI, 95% confidence interval.

Fermented dairy product as the sum of yogurt and cheese intake; Non-fermented dairy products correspond to the milk food item.

Bold values are those statistically significant (P-value<0.005).

## 4 Discussion

The current study investigated the association between dairy product intake and frailty transitions in a cohort of Italian community-dwelling adults older than 65 years enrolled in the InCHIANTI study. According to our findings, this population's habitual consumption of total dairy products was not significantly associated with frailty transitions’ probabilities during the 9 years of follow-up. Some previous studies have assessed the association between dairy products and frailty risk, obtaining inconclusive results [[25], [26], [27], [28], [29],[40], [41], [42], [43]]. A recent prospective analysis of the Nurse’s Health Study found similar intakes of dairy products as our study: 0.83 servings of milk, 0.13 of yogurt, and 0.56 of cheese per day. However, they showed that women with an intake of cheese ≥ 1 serving per day had a significantly increased risk of frailty (RR = 1.17, 95% CI: 1.07, 1.28) (*p* < 0.001) 29]. While, an analysis performed by Rahi et al., found null results when evaluating the cross-sectional and prospective associations between the intake of dairy products (including total dairy, milk, fresh dairy, and cheese) and frailty risk after 10 years of follow-up in a French cohort of older adults [26]. Likewise, O’Connell et al. observed no association between milk and dairy consumption and physical frailty risk in an Irish Community-Dwelling Older Adults cohort [17]. Similarly, another observational study on older adults from the Korean Frailty and the Aging Cohort Study showed that the “milk” pattern was not associated with the frailty status [44]. Other results from the Nurse’s Health Study also found no significant association between protein intake from dairy products and the frailty risk in fully adjusted models 40].

On the contrary, Otsuka et al. observed that consumption of 161.5 g per day of milk and dairy products was positively associated with the frailty transition from prefrail to robust among 130 older Japanese participants of the Longevity Sciences-Longitudinal Study of Aging [41]. Moreover, Lana et al. found that participants consuming ≥7 servings per week of low-fat milk and yogurt had a lower risk of frailty at 3.5 years of follow-up (OR = 0.52, 95% CI: 0.29−0.90) compared to those consuming <1 serving per week in a Spanish population aged ≥60 years [25]. Likewise, a study based on Japanese older adults observed that a high dairy intake was negatively associated with the frailty development (OR = 0.73, 95% CI: 0.55−0.96) after 2 years of follow-up [41]. Similarly, an analysis from the Framingham Heart Study (*n* = 2554 men and women) showed an inverse association between yogurt intake and the odds of frailty onset [28]. In the same way, our result shows that a higher yogurt intake was associated with a greater probability to reverse from frail to pre-frail status, which supports a potential beneficial effect of yogurt intake. However, our statistical power analysis may have affected this finding because the percentage of yogurt consumers was low, and the probability for the transition from frail to pre-frail was nearly 18% between different evaluations. Nevertheless, our study offers an interesting insight into the current literature on this topic. It underlines that the potential beneficial effect of certain dietary products may vary based on the baseline frailty status.

Dairy products are one of the main sources of protein intake in older people [[43], [44], [45], [46]], and it is well known that adequate protein intake can induce a positive protein balance, stimulating muscle protein synthesis and inhibiting protein breakdown [45]. Indeed, a systematic review [27] concluded that consuming dairy products in older people could reduce the frailty risk, considering a high intake of low-fat milk and yogurt. In addition, they suggested that including nutrient-rich dairy proteins in the usual diet (*e.g.*, ricotta cheese) may reduce the risk of sarcopenia by improving skeletal muscle mass. Fermented dairy products, and especially yogurt, are considered nutrient-dense foods with good digestibility and an important source of probiotics, which may a) modulate gut microbiota, b) increase mineral absorption, and c) positively affect immune system and metabolic pathways, all of which could have a role in lowering frailty risk [[46], [47], [48]]. Indeed, several studies have proposed that nutrients, such as vitamin K from fermented dairy products, could act as a cofactor in osteocalcin synthesis, favoring the bone mineralization process [49]. Thus, a higher intake of dairy products could decrease the risk of frailty by delaying sarcopenia and bone mass loss, although further research is needed to elucidate possible mechanisms. Another possible explanation for our results could be related to social factors associated with the consumption of dairy products. One could hypothesize that frail individuals with greater assistance from formal or informal caregivers have more chances to keep a healthy dietary style due to factors like masticatory difficulties or edentulism that are common in frail subjects and can severely impact physical and muscle health [50,51]. Moreover, as largely demonstrated, closer assistance to older frail individuals can also positively promote physical activities and social life, with an overall benefit on psychological well-being [52].

Strengths of this research are the size of the cohort studied with a follow-up over 9 years, the use of a validated FFQ to estimate the habitual intake of foods and nutrients, and the availability of repeated measures in the analysis for dairy product consumption and frailty. This is very relevant since older people, even frail ones, are susceptible to change their dietary habits over time due to different factors that affect their health status (physiologic, pathological, and/or psychologic factors) [53]. Among the main limitations, a part of the potential measurement error, are the possible recall bias in the self-reported dietary assessment and the lack of data concerning the type of dairy products (high-fat *vs* low-fat) in the FFQ. Therefore, we cannot investigate if low-fat dairy products which are recommended in the current dietary guidelines are more relevant for reducing frailty risk. Lastly, frail to pre-frail transitions in our study (18%) were lower than what was reported in previous systematic reviews (around 35–40 %) [54,55]. This fact, most likely attributable to the mean age at baseline of our population (mean age 74 years old), could have biased our analyses. Although the calculated models were robust, not all the transitions could be computed, for example frail to robust. Moreover, the literature suggest that improvements in frailty are less frequent in high-income countries, especially in the US, compared to other geographical areas such as China and South America [54,55]. As the consumption of dairy products, our study is also more comparable with the US [29] than other areas, especially Asia [56]; therefore, the generalizability of our results is probably limited to high-income countries in Europe and North America.

## 5 Conclusion

In conclusion, this study suggests that consuming dairy products does not play a strong role in preventing frailty development in older persons living in a Mediterranean country. However, some dairy products, especially yogurt, could promote frailty reversion due to their nutrient-dense nature, providing high-quality protein, micronutrients, and bioactive compounds. Moreover, including dairy products in an older person’s diet is practical and convenient, especially if they have a consistency that could overcome some barriers in food consumption, like tooth loss and chewing difficulties. Most of the current dietary guidelines recommend the consumption of 3 servings per day of low-fat dairy products in adults, including in older adults [21]. Our findings are in line with these public health recommendations for community-dwellings older adults, highlighting the potential key role of yogurt compared to other dairy products. Nevertheless, gfurther studies are needed to enhance the scientific evidence about this topic, differentiating between low and high-fat dairy products and investigating populations from different geographical areas considering baseline frailty status and its transitions over time.

## CRediT authorship contribution statement

RZ-R and CA-L designed the research; NHL, TM and CT performed the statistical analyses; NHL wrote the draft; TM, RZ-R, AC, CT, MF, SB, LF and CA-L provided critical revision; AC, MF, SB and LF provided data of the study; CA-L had primary responsibility for final content. All authors read and approved the final manuscript.

## Funding

The InCHIANTI study was partly supported by the Italian Ministry of Health and by the 10.13039/100000049U.S. National Institute on Aging (N.I.A).

This study was funded by 10.13039/501100011584Danone Institute and further supported by the Instituto de Salud Carlos III through the project CIBERFES, CB16/10/00269, and the grant PID2020-114921RB-C21, PID2021-128542OA-I00 and PID2023-148013OB-C21, and Maria de Maeztu Unit of Excellence grant (CEX2021-001234-M) funded by MCIU/AEI/ Co-funded by European Regional Development Fund, ERDF, a way to build Europe. The Generalitat de Catalunya’s Agency AGAUR of 2021SGR00687 and ICREA Academia. IDIBELL is a member of the CERCA Programme, Generalitat de Catalunya. This article was also developed within the project funded by Next Generation EU - “Age-It - ageing well in an ageing society” project (PE0000015), National Recovery and Resilience Plan (NRRP) - PE8 - Mission 4, C2, Intervention 1.3”. The views and opinions expressed are only those of the authors and do not necessarily reflect those of the European Union or the European Commission. Neither the European Union nor the European Commission can be held responsible for them.

## Data availability

Data is available to all researchers upon justified request using the proposal form on the InCHIANTI website (https://www.nia.nih.gov/inchianti-study).

## Declaration of competing interest

The authors declare that they have no conflict of interests.

## References

[1] Fried L.P., Tangen C.M., Walston J., Newman A.B., Hirsch C., Gottdiener J. (2001). Frailty in older adults: evidence for a phenotype. J Gerontol A Biol Sci Med Sci.

[2] Clegg A., Young J., Iliffe S., Rikkert M.O., Rockwood K. (2013). Frailty in elderly people. Lancet.

[3] Dent E., Morley J.E., Cruz-Jentoft A.J., Woodhouse L., Rodríguez-Mañas L., Fried L.P. (2019). Physical frailty: ICFSR International clinical practice guidelines for identification and management. J Nutr Health Aging.

[4] World Health Organization (2015).

[5] Dent E., Kowal P., Hoogendijk E.O. (2016). Frailty measurement in research and clinical practice: a review. Eur J Intern Med.

[6] Yannakoulia M., Ntanasi E., Anastasiou C.A., Scarmeas N. (2017). Frailty and nutrition: from epidemiological and clinical evidence to potential mechanisms. Metabolism.

[7] Bonnefoy M., Berrut G., Lesourd B., Ferry M., Gilbert T., Guerin O. (2015). Frailty and nutrition: searching for evidence. J Nutr Health Aging.

[8] Lorenzo-López L., Maseda A., de Labra C., Regueiro-Folgueira L., Rodríguez-Villamil J.L., Millán-Calenti J.C. (2017). Nutritional determinants of frailty in older adults: a systematic review. BMC Geriatr.

[9] Ble A., Cherubini A., Volpato S., Bartali B., Walston J.D., Windham B.G. (2006). Lower plasma vitamin E levels are associated with the frailty syndrome: the InCHIANTI study. J Gerontol A Biol Sci Med Sci.

[10] Bartali B., Frongillo E.A., Bandinelli S., Lauretani F., Semba R.D., Fried L.P. (2006). Low nutrient intake is an essential component of frailty in older persons. J Gerontol A Biol Sci Med Sci.

[11] Smit E., Winters-Stone K.M., Loprinzi P.D., Tang A.M., Crespo C.J. (2013). Lower nutritional status and higher food insufficiency in frail older US adults. Br J Nutr.

[12] Coelho-Júnior H.J., Rodrigues B., Uchida M., Marzetti E. (2018). Low protein intake is associated with frailty in older adults: a systematic review and meta-analysis of observational studies. Nutrients.

[13] Cella A., Veronese N., Poli S., Custureri R., Delrio A., Musacchio C. (2019). Higher animal-derived dietary protein intake is associated with lower prevalence of frailty. Int J Gerontol.

[14] Struijk E.A., Rodriguez-Artalejo F., Fung T.T., Willett W.C., Hu F.B., Lopez-Garcia E. (2020). Sweetened beverages and risk of frailty among older women in the Nurses’ Health Study: a cohort study. PLoS Med.

[15] García-Esquinas E., Rahi B., Peres K., Colpo M., Dartigues J.F., Bandinelli S. (2016). Consumption of fruit and vegetables and risk of frailty: a dose-response analysis of 3 prospective cohorts of community-dwelling older adults. Am J Clin Nutr.

[16] Kojima G., Avgerinou C., Iliffe S., Jivraj S., Sekiguchi K., Walters K. (2018). Fruit and vegetable consumption and frailty: a systematic review. J Nutr Health Aging.

[17] O’Connell M.L., Coppinger T., Lacey S., Walton J., Arsenic T., McCarthy A.L. (2021). Associations between food group intake and physical frailty in Irish community-dwelling older adults. Nutr Metab Insights.

[18] Rashidi Pour Fard N., Amirabdollahian F., Haghighatdoost F. (2019). Dietary patterns and frailty: a systematic review and meta-analysis. Nutr Rev.

[19] Watanabe D., Kurotani K., Yoshida T., Nanri H., Watanabe Y., Date H. (2022). Diet quality and physical or comprehensive frailty among older adults. Eur J Nutr.

[20] Zhang K., Wu J. (2024). Meat-egg-dairy consumption and frailty among Chinese older adults: exploring rural/urban and gender differences. Nutrients.

[21] (2020). U.S. Department of Agriculture and U.S. Department of Health and Human Services. Dietary guidelines for Americans, 2020-2025.

[22] Mozaffarian D. (2019). Dairy foods, obesity, and metabolic health: the role of the food matrix compared with single nutrients. Adv Nutr.

[23] Papadimitriou N., Bouras E., van den Brandt P.A., Muller D.C., Papadopoulou A., Heath A.K. (2022). A prospective diet-wide association study for risk of colorectal cancer in EPIC. Clin Gastroenterol Hepatol.

[24] Dehghan M., Mente A., Rangarajan S., Sheridan P., Mohan V., Iqbal R. (2018). Association of dairy intake with cardiovascular disease and mortality in 21 countries from five continents (PURE): a prospective cohort study. Lancet.

[25] Lana A., Rodriguez-Artalejo F., Lopez-Garcia E. (2015). Dairy consumption and risk of frailty in older adults: a prospective cohort study. J Am Geriatr Soc.

[26] Rahi B., Pellay H., Chuy V., Helmer C., Samieri C., Féart C. (2021). Dairy product intake and long-term risk for frailty among french elderly community dwellers. Nutrients.

[27] Cuesta-Triana F., Verdejo-Bravo C., Fernández-Pérez C., Martín-Sánchez F.J. (2019). Effect of milk and other dairy products on the risk of frailty, sarcopenia, and cognitive performance decline in the elderly: a systematic review. Adv Nutr.

[28] Siefkas A.C., Millar C.L., Dufour A.B., Kiel D.P., Jacques P.F., Hannan M.T. (2022). Dairy food intake is not associated with frailty in adults from the framingham heart study. J Acad Nutr Diet.

[29] Struijk E.A., Fung T.T., Rodriguez-Artalejo F., Bischoff-Ferrari H.A., Willett W.C., Lopez-Garcia E. (2024). Specific dairy foods and risk of frailty in older women: a prospective cohort study. BMC Med.

[30] Ratajczak A.E., Zawada A., Rychter A.M., Dobrowolska A., Krela-Kaźmierczak I. (2021). Milk and dairy products: Good or Bad for human bone? Practical dietary recommendations for the prevention and management of osteoporosis. Nutrients.

[31] Hanach N.I., McCullough F., Avery A. (2019). The impact of dairy protein intake on muscle mass, muscle strength, and physical performance in middle-aged to older adults with or without existing sarcopenia: a systematic review and meta-analysis. Adv Nutr.

[32] Greco E.A., Pietschmann P., Migliaccio S. (2019). Osteoporosis and sarcopenia increase frailty syndrome in the elderly. Front Endocrinol (Lausanne).

[33] Lachat C., Hawwash D., Ocké M.C., Berg C., Forsum E., Hörnell A. (2016). Strengthening the reporting of observational studies in epidemiology—nutritional epidemiology (STROBE-nut): an extension of the STROBE statement. PLoS Med.

[34] Ferrucci L., Bandinelli S., Benvenuti E., Di Iorio A., Macchi C., Harris T.B. (2000). Subsystems contributing to the decline in ability to walk: bridging the gap between epidemiology and geriatric practice in the InCHIANTI study. J Am Geriatr Soc.

[35] Pisani P., Faggiano F., Krogh V., Palli D., Vineis P., Berrino F. (1997). Relative validity and reproducibility of a food frequency dietary questionnaire for use in the Italian EPIC centres. Int J Epidemiol.

[36] Elosua R., Bartali B., Ordovas J.M., Corsi A.M., Lauretani F., Ferrucci L. (2005). Association between physical activity, physical performance, and inflammatory biomarkers in an elderly population: the InCHIANTI study. J Gerontol A Biol Sci Med Sci.

[37] Fava G.A. (1980). Assessing depressive symptoms across cultures: Italian validation of the CES-D self-rating scale. J Clin Psychol.

[38] Kragh Andersen P., Pohar Perme M., van Houwelingen H.C., Cook R.J., Joly P., Martinussen T. (2021). Analysis of time-to-event for observational studies: guidance to the use of intensity models. Stat Med.

[39] Vansteelandt S. (2012). Dealing with complex problems of confounding in mediation analysis. 46th scientific meeting of the Italian Statistical Society.

[40] Struijk E.A., Fung T.T., Rodríguez-Artalejo F., Bischoff-Ferrari H.A., Hu F.B., Willett W.C. (2022). Protein intake and risk of frailty among older women in the Nurses’ Health Study. J Cachexia Sarcopenia Muscle.

[41] Otsuka R., Tange C., Tomida M., Nishita Y., Kato Y., Yuki A. (2019). Dietary factors associated with the development of physical frailty in community-dwelling older adults. J Nutr Health Aging.

[42] Hong Y.J., Otsuka R., Song Z., Fukuda C., Tajima R., Lin J. (2024). Association between milk consumption in middle age and frailty in later life: the Aichi Workers’ cohort study. Geriatr Gerontol Int.

[43] Otsuka R., Zhang S., Tange C., Nishita Y., Tomida M., Kinoshita K. (2022). Association of dietary intake with the transitions of frailty among Japanese community-dwelling older adults. J Frailty Aging.

[44] Kim J., Lee Y., Won C.W., Kim M.K., Kye S., Shim J.S. (2021). Dietary patterns and frailty in older Korean adults: results from the Korean frailty and aging cohort study. Nutrients.

[45] Boirie Y. (2009). Physiopathological mechanism of sarcopenia. J Nutr Health Aging.

[46] Biver E., Durosier-Izart C., Merminod F., Chevalley T., van Rietbergen B., Ferrari S.L. (2018). Fermented dairy products consumption is associated with attenuated cortical bone loss independently of total calcium, protein, and energy intakes in healthy postmenopausal women. Osteoporos Int.

[47] Rizzoli R., Biver E. (2018). Effects of fermented milk products on bone. Calcif Tissue Int.

[48] Lim M.Y., Hong S., Kim J.H., Nam Y.-D. (2021). Association between gut microbiome and frailty in the older adult population in Korea. J Gerontol A Biol Sci Med Sci.

[49] Van Ballegooijen A.J., Pilz S., Tomaschitz A., Grübler M.R., Verheyen N. (2017). The synergistic interplay between vitamins D and K for bone and cardiovascular health: a narrative review. Int J Endocrinol.

[50] Velázquez-Olmedo L.B., Borges-Yáñez S.A., Andrade Palos P., García-Peña C., Gutiérrez-Robledo L.M., Sánchez-García S. (2021). Oral health condition and development of frailty over a 12-month period in community-dwelling older adults. BMC Oral Health.

[51] Musacchio E., Binotto P., Perissinotto E., Sergi G., Zambon S., Corti M.C. (2021). Tooth retention predicts good physical performance in older adults. PLoS One.

[52] Böhm A.W., Mielke G.I., Da Cruz M.F., Viana Ramires V., Wehrmeister F.C. (2016). Social support and leisure-time physical activity among the elderly: a population-based study. J Phys Act Health.

[53] Jyväkorpi S.K., Ramel A., Strandberg T.E., Piotrowicz K., Błaszczyk-Bębenek E., Urtamo A. (2021). The sarcopenia and physical frailty in older people: multi-component treatment strategies (SPRINTT) project: description and feasibility of a nutrition intervention in community-dwelling older Europeans. Eur Geriatr Med.

[54] Kojima G., Taniguchi Y., Iliffe S., Jivraj S., Walters K. (2019). Transitions between frailty states among community-dwelling older people: a systematic review and meta-analysis. Ageing Res Rev.

[55] Ofori-Asenso R., Chin K.L., Mazidi M., Zomer E., Ilomaki J., Ademi Z. (2020). Natural regression of frailty among Community-dwelling older adults: a systematic review and meta-analysis. Gerontologist.

[56] Chen A., Moradi S., Huang J., Xu S., Sismey M., Hort J. (2024). Older Chinese adults’ milk consumption habits: a study across 5 cities. J Dairy Sci.

